# Occult Macular Dystrophy

**DOI:** 10.4274/tjo.26234

**Published:** 2016-04-05

**Authors:** Işıl Sayman Muslubaş, Serra Arf, Mümin Hocaoğlu, Hakan Özdemir, Murat Karaçorlu

**Affiliations:** 1 İstanbul Retina Institute, İstanbul, Turkey

**Keywords:** Occult macular dystrophy, optical coherence tomography, microperimetry, multifocal electroretinogram

## Abstract

Occult macular dystrophy is an inherited macular dystrophy characterized by a progressive decline of bilateral visual acuity with normal fundus appearance, fluorescein angiogram and full-field electroretinogram. This case report presents a 20-year-old female patient with bilateral progressive decline of visual acuity for six years. Her visual acuity was 3-4/10 in both eyes. Anterior segment and fundus examination, fluorescein angiogram and full-field electroretinogram were normal. She could read all Ishihara pseudoisochromatic plates. Fundus autofluorescence imaging was normal. There was a mild central hyporeflectance on fundus infrared reflectance imaging in both eyes. Reduced foveal thickness and alterations of the photoreceptor inner and outer segment junction were observed by optical coherence tomography in both eyes. Central scotoma was also found by microperimetry and reduced central response was revealed by multifocal electroretinogram in both eyes. These findings are consistent with the clinical characteristics of occult macular dystrophy.

## INTRODUCTION

Occult macular dystrophy (OMD) is an inherited macular dystrophy characterized by progressive bilateral vision loss despite normal fundus appearance.^1^ Fundus fluorescein angiogram (FFA) and full-field electroretinogram (ERG) are normal, whereas results of multifocal ERG (mfERG) of the central retina are markedly reduced.^1,2^ Foveal thinning and disruptions of the photoreceptor inner and outer segment (IS/OS) junction are observed on spectral domain optical coherence tomography (SD-OCT). In many patients, disruptions of the photoreceptor layer detected by SD-OCT are correlated with visual acuity and disease progression.^3^ Microperimetry (MP) is a visual field technique used in macular diseases to determine retinal sensitivity.^4^ The method was developed to identify fixation alterations due to scotoma and vision loss in conditions involving the central retina, and can be utilized for this purpose in OMD patients.^5,6,7^ On fundus infrared reflectance (IR) imaging, OMD patients exhibit central hyporeflectance which becomes more pronounced with disease progression.^8^ Abnormalities are not apparent on fundus autofluorescence (FAF) imaging in most OMD patients, although mild central hyperautofluorescence can be observed in a minority of patients.^8,9^

In this case report, we aimed to present the clinical characteristics and diagnostic methods of a patient we diagnosed with OMD.

## CASE REPORT

**Figure 1:**
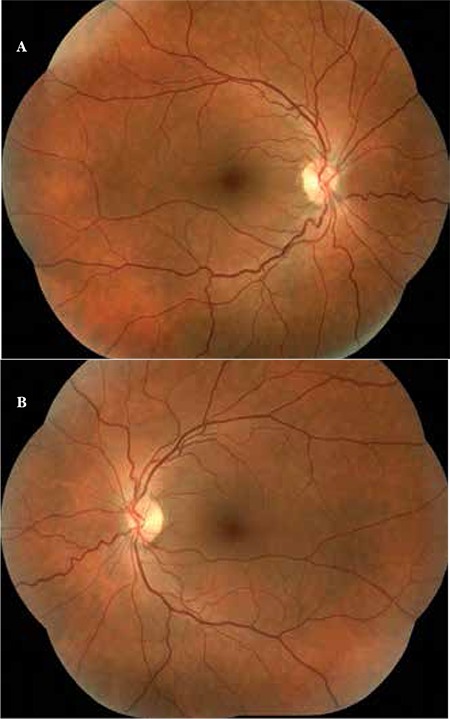
Fundus color image from right eye (A) and left eye (B)

**Figure 2:**
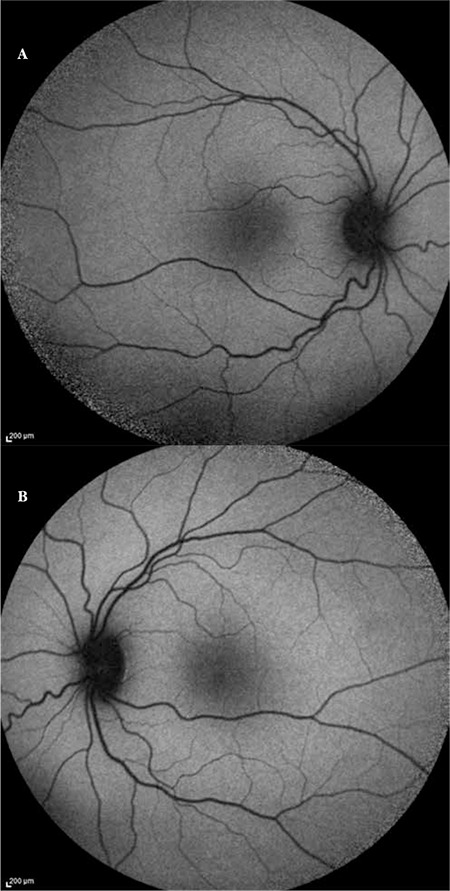
Fundus autofluorescence image from right eye (A) and left eye (B)

**Figure 3:**
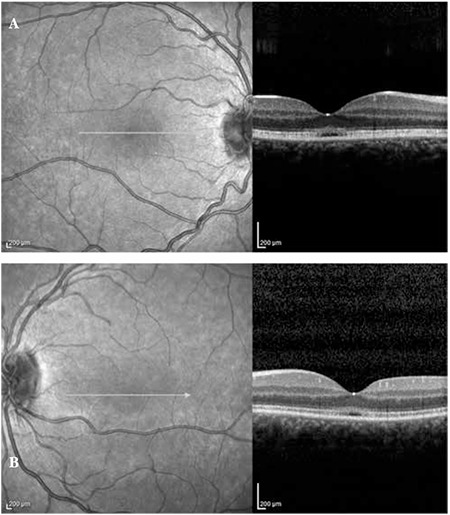
Infrared reflectance image from right eye (A) and left eye (B)

**Figure 4:**
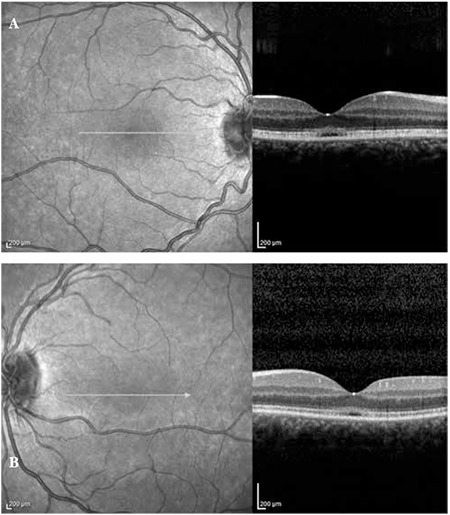
Optical coherence tomography image from right eye (A) and left eye (B)

**Figure 5:**
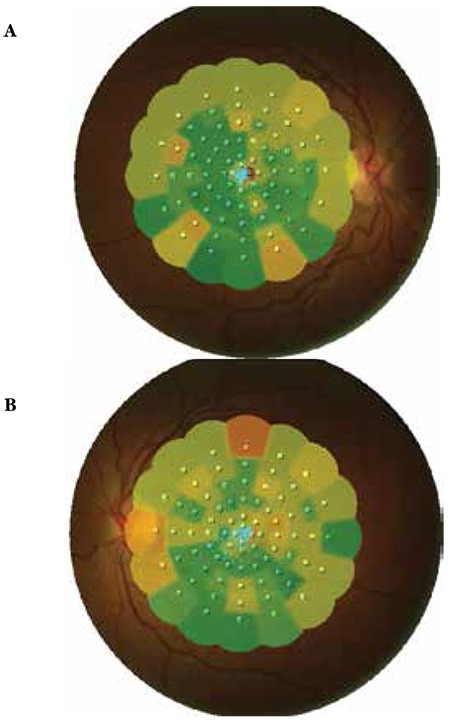
Microperimetry from right eye (A) and left eye (B)

**Figure 6:**
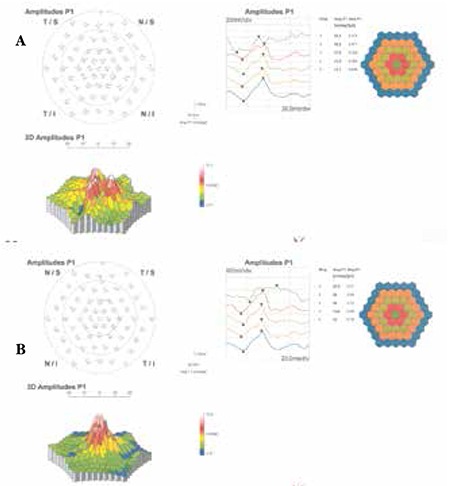
Multifocal electroretinogram from right eye (A) and left eye (B)

A 20-year-old female patient with a 6-year history of progressive bilateral vision loss was referred to our clinic. The patient had no known systemic disease, previous trauma, family history, history of drug or cigarette use, or consanguineous marriage in her family. Her visual acuity was 3-4/10 in both eyes; intraocular pressure was 11 mmHg in the right eye and 13 mmHg in the left eye. No pathologies were detected during anterior segment examination. Fundus examination revealed no pathologies other than mild retinal vessel tortuosity (Figure 1A and 1B). Both eyes appeared normal on FAF imaging (Figure 2A and 2B). Mild central hyporeflectance was observed in both eyes on fundus IR imaging (Figure 3A and 3B). FFA was normal. Foveal thickness was determined by OCT thickness profile analysis as 155 µm and 188 µm in the right and left eyes, respectively. Disruption of the photoreceptor IS/OS junction was observed. The extension of the IS/OS band disruption on the horizontal axis was measured as 696 µm in the right and 348 µm in the left eye (Figure 4A and 4B). On MP analysis both eyes exhibited relatively unstable fixation which was more pronounced in the right eye, and areas of absolute scotoma consistent with OCT were observed. Retinal sensitivity in the central 20° field of the macula was measured as 13.9 dB in the right and 13.5 dB in the left eye (Figure 5A and 5B). Full-field ERG was normal, but mfERG revealed a bilateral reduction in central response which was more pronounced in the right eye (Figure 6A and 6B).

## DISCUSSION

OMD is an inherited macular dystrophy, called occult because the fundus appears normal despite macular dysfunction. OMD was first described by Miyake et al.^1^ and although it is autosomal dominantly inherited, sporadic cases have also been reported.

Many studies have reported that the age at onset for OMD ranges widely, from 6 to 81 years.^9^ In our case, the patient’s vision loss began at age 14 and she was diagnosed at age 20.

As OMD is a central retinal disease, patients’ full-field ERG results are normal, while responses in focal macular ERG and mfERG are markedly reduced.^1,2^ In addition, measuring central retinal sensitivity by MP may reveal scotoma or fixation loss in OMD patients.5 As described in the literature, our patient had normal full-field ERG results, but on mfERG she exhibited a bilateral reduction in central response that was more pronounced in the right eye. On MP we detected relatively unstable fixation in both eyes which was also more pronounced in the right eye. Reduced retinal sensitivity was observed in the central 8° field of the maculae of both eyes.

Structural changes in the photoreceptor layer in OMD patients are easily detected by SD-OCT. Many studies using SD-OCT imaging have reported pronounced photoreceptor damage in the fovea, reduced foveal thickness and disruptions of the photoreceptor IS/OS junction in OMD patients.^3^

It has been demonstrated that the severity of photoreceptor layer disruption is correlated with visual acuity and disease progression.^3^ Similarly, in our patient we observed bilateral foveal thinning and disruption of the photoreceptor IS/OS junction, both of which were more pronounced in the right eye.

Because fundus IR imaging is easily performed and reveals central hyporeflectance in OMD patients which becomes more apparent as the disease progresses, it can be utilized as an auxiliary diagnostic method.^8,9,10,11^ In OMD, no discernible abnormality can be detected by FAF because the condition primarily affects the photoreceptors and there is no evident damage to the retinal pigment epithelium.^9^ Our patient also appeared normal on FAF, while mild central hyporeflectance was observed on fundus IR imaging.

## CONCLUSION

In summary, for patients with progressive vision loss and normal fundus appearance and FFA clinically consistent with OMD, SD-OCT is a primary tool which is non-invasive, easily performed, and clinically reliable. Fundus IR, mfERG and MP are other auxiliary diagnostic methods.

## Ethics

Informed Consent: In accordance with the principles of the Declaration of Helsinki, patients were informed about their current status and natural course, consent was obtained.

Peer-review: Externally peer-reviewed.
